# *ABCC6* knockdown in HepG2 cells induces a senescent-like cell phenotype

**DOI:** 10.1186/s11658-017-0036-2

**Published:** 2017-04-04

**Authors:** Rocchina Miglionico, Angela Ostuni, Maria Francesca Armentano, Luigi Milella, Elvira Crescenzi, Monica Carmosino, Faustino Bisaccia

**Affiliations:** 1 Department of Sciences, University of Basilicata, Via dell’Ateneo Lucano, 85100 Potenza, Italy; 2 Institute of Experimental Endocrinology and Oncology (IEOS), National Research Council (CNR), 80131 Naples, Italy

**Keywords:** ABCC6, Cell cycle, Senescence, Reductive stress

## Abstract

**Background:**

Pseudoxanthoma elasticum (PXE) is characterized by progressive ectopic mineralization of elastic fibers in dermal, ocular and vascular tissues. No effective treatment exists. It is caused by inactivating mutations in the gene encoding for the ATP-binding cassette, sub-family C member 6 transporter (ABCC6), which is mainly expressed in the liver. The ABCC6 substrate (s) and the PXE pathomechanism remain unknown. Recent studies have shown that overexpression of ABCC6 in HEK293 cells results in efflux of ATP, which is rapidly converted into nucleoside monophosphates and pyrophosphate (PPi). Since the latter inhibits mineralization, it was proposed that the absence of circulating PPi in PXE patients results in the characteristic ectopic mineralization. These studies also demonstrated that the presence of ABCC6 modifies cell secretory activity and suggested that ABCC6 can change the cell phenotype.

**Methods:**

Stable *ABCC6* knockdown HepG2 clones were generated using small hairpin RNA (shRNA) technology. The intracellular glutathione and ROS levels were determined. Experiments using cell cycle analysis, real-time PCR and western blot were performed on genes involved in the senescence phenotype.

**Results:**

To shed light on the physiological role of ABCC6, we focused on the phenotype of HepG2 cells that lack ABCC6 activity. Interestingly, we found that *ABCC6* knockdown HepG2 cells show: 1) intracellular reductive stress; 2) cell cycle arrest in G1 phase; 3) upregulation of p21^Cip^ p53 independent; and 4) downregulation of lamin A/C.

**Conclusions:**

These findings show that the absence of ABCC6 profoundly changes the HepG2 phenotype, suggesting that the PXE syndrome is a complex metabolic disease that is not exclusively related to the absence of pyrophosphate in the bloodstream.

## Background

ATP-binding cassette sub-family C member 6 transporter (ABCC6) is an ATP-dependent transporter mainly found in the basolateral plasma membrane of hepatic and kidney cells. Mutations in the gene were associated with pseudoxanthoma elasticum (PXE), an autosomal recessive disease characterized by progressive ectopic calcification of elastic fibers in dermal, ocular and vascular tissues [1–3]. It is currently known that mutations in the *ABCC6* gene are associated with some cases of generalized arterial calcification of infancy (GACI) [4] and are also responsible for dystrophic cardiac calcification (DCC) in mice, an autosomal recessive trait characterized by calcium phosphate deposits in myocardial tissue [5–7]. Arterial calcification due to CD73 deficiency (ACDC) is a closely related ectopic disorder that probably shares a common pathogenetic pathway with PXE [8].

One early surprising observation was that *ABCC6* is primarily expressed in the liver, to a lesser extent in the proximal tubules of the kidneys, and at very low level, if at all, in tissues clinically affected by PXE [9, 10]. These observations raised a challenging question on the pathomechanism of PXE. The “metabolic hypothesis” postulates that the absence of ABCC6 activity in the liver results in a deficiency of circulating factors that are physiologically required to prevent aberrant mineralization in the connective tissues under normal calcium and phosphate homeostatic conditions [11]. Therefore, in recent years, particular attention has been paid to the analysis of serum and fibroblasts from PXE patients [12, 13]. Proteomics and metabolomics studies on PXE patient fibroblasts revealed increased oxidative stress parameters in these cells [14].

Although ABCC6 is not an ATP transporter [15], recent studies have shown that the overexpression of ABCC6 in HEK293 cells results in the efflux of ATP and other nucleoside triphosphates, which are then rapidly converted into nucleoside monophosphates and pyrophosphate (PPi). Since PPi inhibits mineralization, it was proposed that the absence of circulating PPi in PXE patients results in the characteristic ectopic mineralization of PXE [15, 16]. Notably, these findings also demonstrate that the presence of ABCC6 modifies the cell secretory activity, suggesting that ABCC6 can change the cell phenotype.

However, the role of ABCC6 in the liver has been poorly investigated thus far. To shed light on its physiological role, several structural studies on its domains were performed [17–19]. Here, we focused our attention on the phenotype of HepG2 cells that lack ABCC6 activity. Our previous studies of *ABCC6* knockdown HepG2 cells showed dysregulation of some genes related with the calcification processes, such as the tissue nonspecific alkaline phosphatase (TNAP), the cluster of differentiation 73 (CD73) and osteopontin (OPN) [20]. Importantly, the pro-mineralization genes dysregulated in *ABCC6* knockdown HepG2 cells encode both the proteins expressed on the extracellular face of the hepatocyte plasma membrane (TNAP, CD73) and those released into the bloodstream (OPN, Fetuin A). TNAP and CD73 can exert their activities in the extracellular space and in extra-hepatic tissues, respectively, in the absence of clinically evident modifications in hepatocyte metabolism or function.

In an attempt to shed further light on the functional role of ABCC6 in the liver and on the complex pathogenesis of PXE, we focused on characterizing the cellular phenotype of *ABCC6* knockdown HepG2 cells. We found that these cells show typical features of replicative senescence and the reductive state, suggesting novel and important aspects in the pathophysiological role of ABCC6 in the PXE.

## Methods

### Cell culture and generation of stable shRNA *ABCC6* knockdown clones

HepG2 cells were maintained in Dulbecco’s modified essential medium (DMEM) containing 4.5 g/l glucose, supplemented with 10% fetal bovine serum (FBS), 2 mM L-glutamine, 100 U/ml penicillin and 100 μg/ml streptomycin, at 37 °C in an atmosphere of 5% CO_2_. All the experiments were performed using cells at 70% confluence. Stable clones were generated using the *ABCC6*-shRNA targeting sequence 5′-AGCTTAGACGCGAGAGGTCCATCAAGTCA-3′, as previously described [20].

### Measurement of intracellular reduced glutathione

Intracellular reduced glutathione (GSH) and oxidised glutathione (GSSG) levels were measured using a glutathione fluorometric assay kit (BioVision) according to the manufacturer’s protocol. In this assay, o-phthalaldehyde (OPA) reacts with GSH present in the sample emitting a fluorescent signal (ex. 340 nm, em. 420 nm) that is measured using a GLOMAX Multidetection System (Promega). To measure GSSG specifically, a GSH Quencher is added to remove GSH, preventing its reaction with OPA. GSSG is reduced to GSH, which is measured using the preceding method. The levels of intracellular GSH were quantified using a standard curve for known amounts of GSH. The GSH/GSSG ratio was calculated as an indicator of redox balance.

### Determination of intracellular reactive oxygen species

The intracellular levels of reactive oxygen species (ROS) were determined using a cell permeable fluorogenic probe, 2′,7′-dichlorofluorescein diacetate (DCFH-DA). Once inside the cells, it is converted by intracellular esterases into the cell membrane-impermeable non-fluorescent compound DCFH, which rapidly oxidizes, becoming the highly fluorescent compound dichloro-fluorescein (DCF) in the presence of ROS. Briefly, 2 × 10^5^ cells were seeded into 12-well plates and, after 24 h, incubated with 1 μM DCFH-DA for 1 h at 37 °C in a humidified 5% CO_2_ atmosphere. The cells were then trypsinized, washed with PBS and analyzed via FACScan flow cytometry (BD Biosciences) using 488 nm excitation and 530 nm emission wavelengths.

### Cell cycle analysis

Cells were synchronized at the G0 phase by serum deprivation for 24 h, restimulated with 10% serum for 24 h and incubated with 30 μM bromodeoxyuridine (BrdU, Sigma) for 30 min at 37 °C. Cells were fixed with 70% ethanol in PBS, washed twice with PBS and incubated with 2 M HCl for 30 min at room temperature. The cells were then washed twice with PBS, Tween-20 0.1% (PBST) and incubated with anti-BrdU-FITC (BD Biosciences) for 1 h at room temperature. After incubation, the cells were washed with PBS, resuspended in PBS with 5 μg/ml propidium iodide (Sigma-Aldrich) and 50 μg/ml RNase DNase-free (Roche) and incubated at room temperature for 20 min. Cells were analyzed using a C6 Accuri Flow Cytometer (Beckman Coulter, Inc.) and Modfit software (Verity Software House Inc.).

### Senescence-associated β-galactosidase staining assay

Cytochemical staining for senescence-associated β-galactosidase was determined as described by Dimri et al. [21]. Briefly, cells were seeded into 12-well plates (2 × 10^5^ cells/well) and after 24 h, cells were fixed with 2% formaldehyde and 0.2% glutaraldehyde for 7 min at room temperature. After two washes in PBS, the cells were incubated for 2 h at 37 °C with fresh staining solution containing 0.1% of 5-bromo-4-chloro-3-indolyl β-D-galactopyranoside (X-gal), 5 mM potassium ferrocyanide, 5 mM potassium ferricyanide, 150 mM NaCl and 2 mM MgCl_2_ in 40 mM citric acid/sodium phosphate, pH 6.0 (Sigma-Aldrich). Cells were examined under a light microscope and representative fields were photographed using a Nikon Coolpix P6000.

### RT-PCR and real-time PCR

Total RNA was extracted using the GenElute Mammalian Total RNA Miniprep Kit (Sigma-Aldrich) according to the manufacturer’s instructions. The RNA was transcribed to cDNA using oligo (dT) primers and the GeneAmp RNA PCR Core Kit (Applied Biosystems), and the cDNA was amplified via real-time PCR using PowerSYBR Green PCR Master Mix (Promega) on the 7500 Fast Real-Time PCR System (Applied Biosystems).

The following primers were designed with the Allele ID program to span exon–exon junctions eliminating undesirable genomic DNA amplification: p53, forward: 5′-TGAATGAGGCCTTGGAACTC-3′, reverse: 5′-ACTTCAGGTGGCTGGAGTG3-′; p21^Cip^, forward: 5′-CTGTCTTGTACCCTTGTGCCT-3′, reverse: 5′-CGTTTGGAGTGGTAGAAATCTGTC-3′; lamin A/C, forward: 5′-GTGGATGCTGAGAACAGGCT-3′, reverse: 5′-CACGCAGCTCCTCACTGTAG-3′; and β-actin, forward: 5′-CCTGGCACCCAGCACAAT-3′, reverse: 5′-GCCGATCCACACGGAGTACT-3′.

### Western blot analysis

Cells (5 × 10^5^) were lysed in RIPA buffer (0.1% sodium dodecyl sulfate, 1% NP-40 and 0.5% sodium deoxycholate in PBS at pH 7.4) supplemented with a protease and phosphatase inhibitor cocktail. The proteins (100 μg) were loaded onto SDS-PAGE gels and electrophoretically transferred to nitrocellulose membranes (Amersham Bioscience). After blocking with 5% non-fat dry milk in TRIS buffer saline-Tween 20, the blots were incubated overnight at 4 °C with either 1:1000 anti-lamin A/C (Cell Signaling Antibodies) or 1:1000 anti-p21^Cip^ (Abcam) or 1:1000 anti-p53 (Abcam) primary antibodies, and then with appropriate horseradish peroxidase-conjugated secondary antibody. Proteins were detected using Enhanced Chemiluminescence reagents (ECL, Promega) and chemiluminescence was detected with a Chemidoc XRS detection system equipped with Image Lab Software for image acquisition (Bio-Rad). The quantification of protein bands was performed by determining the relative optical density using ImageJ software (National Institutes of Health, Bethesda, MD).

## Results

### *ABCC6* knockdown HepG2 cells accumulate GSH and show a ‘reductive stress’ state

**Fig. 1:**
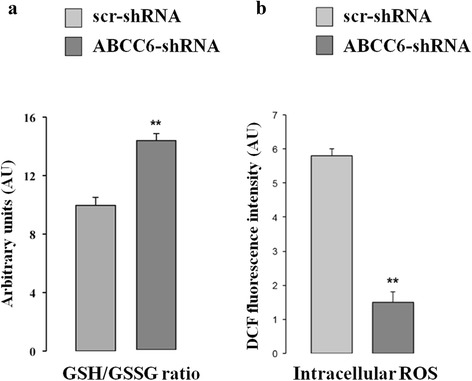
Effect of *ABCC6* knockdown on GSH/GSSG ratio and ROS generation. **a** – The GSH/GSSG ratio was determined using the glutathione fluorometric assay, as described in the Materials and Methods section. The results are reported as means ± SD of three independent experiments performed in triplicate. **b** – ROS production was detected via FACS using a DCFH-DA fluorescent probe as described in the Materials and Methods section. Scr-shRNA: HepG2 cells stably transfected with scrambled shRNA; *ABCC6*-shRNA: HepG2 cells stably transfected with *ABCC6*-shRNA. The values are the means ± SD of three replicates from three independent experiments. **p < 0.01. Unpaired data were assessed for statistical significance using Student’s *t* test

Since an increase in the oxidative state was observed in PXE fibroblasts [14, 22], we first checked whether the knockdown of *ABCC6* in HepG2 cells might affect the intracellular GSH/GSSG ratio. We performed all experiments in stable *ABCC6* knockdown HepG2 cells that had been previously generated and studied [20]. Unexpectedly, as shown in Fig. 1a, the intracellular GSH/GSSG ratio increased by about 40% in *ABCC6* knockdown cells (*ABCC6*-shRNA) compared to control cells stably transfected with scrambled shRNA (scr-shRNA).

Because of the cysteine residue, GSH is readily oxidized non-enzymatically to glutathione disulfide (GSSG) by electrophilic substances (e.g., free radicals and reactive oxygen/nitrogen species), thus effectively scavenging free radicals and other reactive oxygen species (ROS). Accordingly, we found that ROS levels, measured using the fluorescent dye, 2′,7′-dichlorodihydrofluorescein diacetate (H_2_DCF-DA), showed a strong and significant 5-fold decrease in *ABCC6* knockdown cells compared to control HepG2 cells (Fig. 1b). The observed ROS reduction does not depend on DCF efflux mediated by multidrug resistance-associated proteins (MRP) [23]. In fact, the expression of some MRP transporters (ABCC1, ABCC2 and ABCB1), measured using real-time PCR, does not change in ABCC6 knockdown cells compared to control cells (data not shown).

These data suggest that the knockdown of *ABCC6* in HepG2 cells causes the ‘reductive stress’ state [24].

### *ABCC6* knockdown HepG2 cells show impaired cell cycle with cell accumulation in G1 phase

Several studies have suggested the role of intracellular GSH in cell proliferation, showing that each phase in the life of the cells is characterized by a particular redox state, and that proliferating cells are in a mostly reduced state [25, 26].

**Fig. 2:**
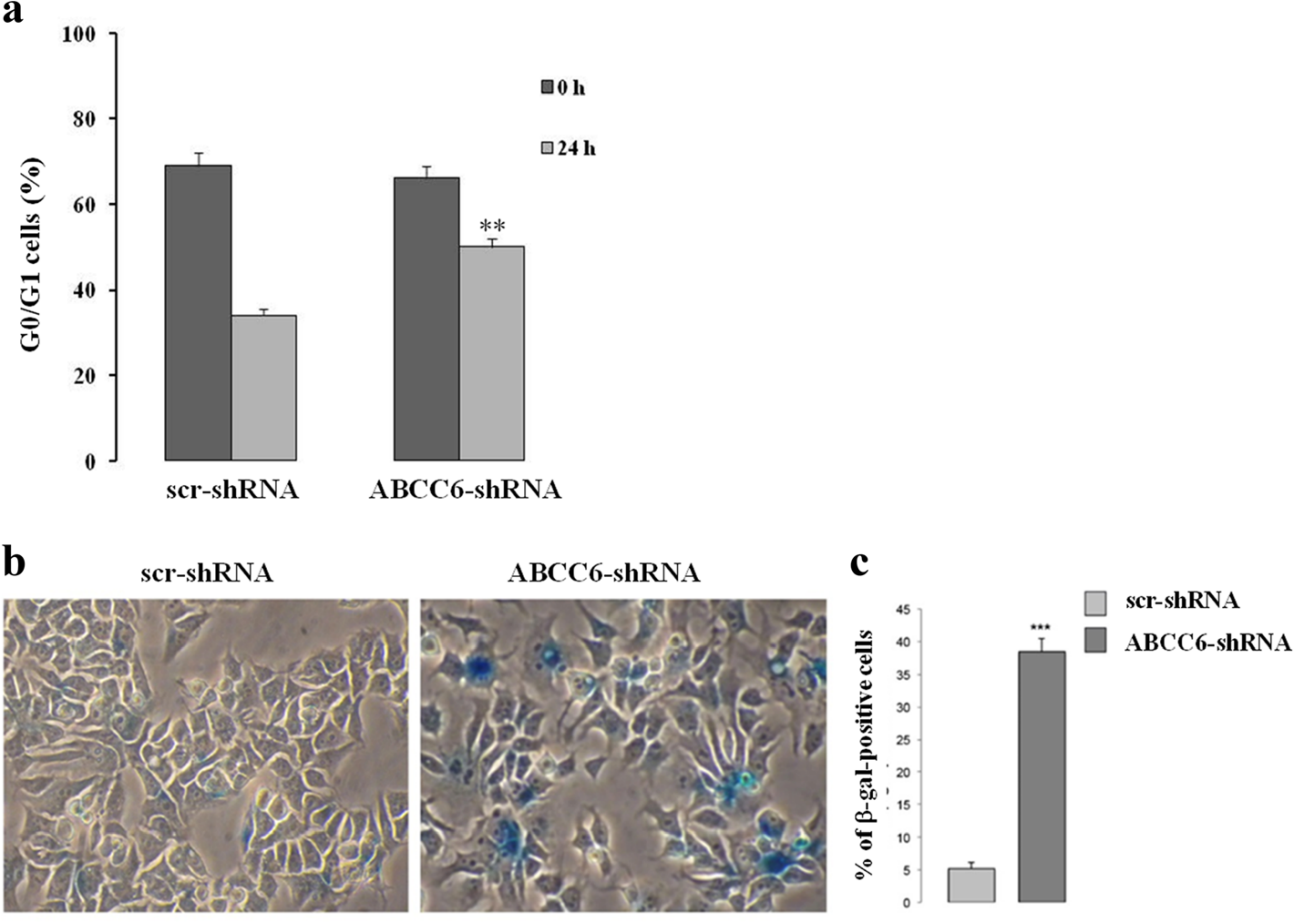
Knockdown of *ABCC6* leads to a senescence-like phenotype. **a** – For the cell cycle analysis, the cells were synchronized at the G1 phase by serum deprivation for 24 h, restimulated with serum for 24 h, and analyzed using flow cytometry after BrDU and PI staining. The percentage of control (scr-shRNA) and *ABCC6* knockdown cells (*ABCC6*-shRNA) in G0/G1 was recorded. The values are the means ± SD of three replicates from three independent experiments. Statistical analysis was performed using unpaired Student’s *t* test: **p < 0.001. **b** – Representative images (40 × magnification) of senescence-associated β-galactosidase staining in control and *ABCC6* knockdown cells. **c** – Quantitative analysis of positive β-galactosidase-stained cells. Data were generated from three independent experiments performed in triplicate and are shown as means ± SD. ***p < 0.001. Unpaired data were assessed for statistical significance using Student’s *t* test

We analyzed the cell cycle in control and *ABCC6* knockdown HepG2 cells. Briefly, 24 h serum starvation was used to synchronize cells in G0 phase. BrdU and PI incorporation were analyzed via flow cytometry 24 h after the addition of serum. We found that *ABCC6* knockdown cells delay G1 exit (Fig. 2a). After 24 h, about 50% of the control cells but only about 20% of *ABCC6* knockdown cells had exited G1, suggesting that the reduced expression of ABCC6 significantly retards G1 to S transition and slows cell growth.

We then analyzed the activity of the well-known cellular senescence biomarker senescence-associated β-galactosidase (SA-β-Gal) at pH 6.0 [27]. As shown in Fig. 2c, *ABCC6* knockdown HepG2 cells show a roughly 8-fold increase in the number of cells with blue precipitates after incubation with the SA-β-gal staining solution, consistent with senescent phenotype. In senescent cells, the cyclin-dependent kinases (CDK) that selectively regulate cell entry into the different cycle phases, are inactivated by CDK inhibitors (CDKI).

**Fig. 3:**
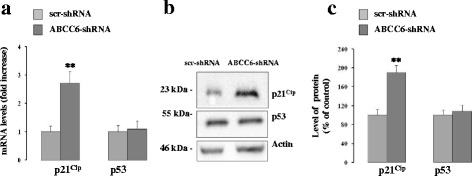
Knockdown of *ABCC6* induces alterations in gene expression. **a** – p21^Cip^ and p53 mRNA levels were quantified with real-time PCR in control (scr-shRNA) and *ABCC6* knockdown cells (*ABCC6*-shRNA), using β-actin as the internal control. Data are expressed as the means ± SD (*n* = 3). **b** – Representative western blots of p21^Cip^ and p53 in control and *ABCC6* knockdown cells. β-actin was used as a loading control. **c** – Densitometric analysis of the immunoreactive bands from three independent experiments (means ± SD). **p < 0.01. Unpaired data were assessed for statistical significance using Student’s *t* test

Fig. 3a shows the increase in p21^Cip^ transcript levels in *ABCC6* knockdown cells compared to control cells, as confirmed by western blotting analysis (Fig. 3b and c), while p53 expression was unaffected by ABCC6 silencing (Fig. 3).

**Fig. 4:**
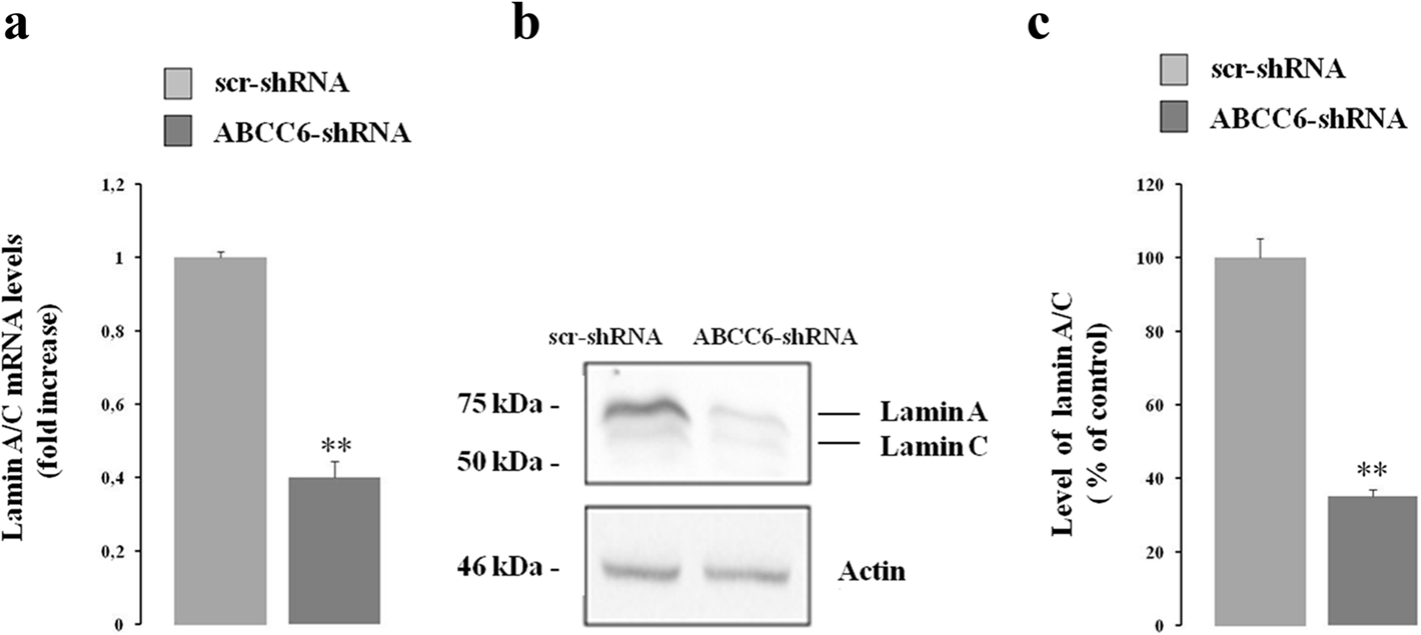
Effect of *ABCC6* knockdown on lamin A/C gene expression. **a** – mRNA levels of lamin A/C were quantified for both control (scr-shRNA) and *ABCC6* knockdown cells (*ABCC6*-shRNA) with real-time PCR using β-actin as the internal control. Data are shown as the means ± SD from three independent experiments. **b** – Representative western blot of lamin A/C in both control and *ABCC6* knockdown cells. β-actin was used as a loading control. **c** – Densitometric analysis of the immunoreactive bands performed in three independent experiments. Data are shown as the means ± SD from three independent experiments. **p < 0.01. Unpaired data were assessed for statistical significance using Student’s *t* test

Since nuclear abnormalities and changes in lamin A/C also occur during the aging process of wild-type cells [28], we monitored the expression levels of both lamin A/C messenger and protein. Real time PCR data show a decrease of about 60% in lamin A/C mRNA in *ABCC6* knockdown cells compared to control cells (Fig. 4a). Western blotting analysis shows a significant decrease in lamin A/C expression in *ABCC6* knockdown cells compared to control cells (Fig. 4b and c).

## Discussion

Previous experiments on PXE dermal fibroblasts have shown an increase in ROS levels that has been suggested to be the cause of major PXE injuries [14, 22]. Unlike the case of fibroblasts, we find that the knockdown of *ABCC6* in HepG2 cells induces a significant increase in the intracellular levels of GSH and a reduction in ROS levels that could reflect the absence of PXE symptoms in the liver. However, antioxidant therapy failed to improve the clinical manifestation of PXE patients [29] and failed to counteract the mineralization in *Abcc6* ^−/−^ mice [30], clearly suggesting that the absence of normal circulating levels of GSH is not the main pathomechanism underlying PXE.

The observed cell cycle delay in the G1 phase could closely correlate to the intracellular ROS reduction, suggesting a functional inextricable relationship between ROS levels and G1/S cell cycle status**,** according to several studies [31, 32].

The change in the intracellular redox state observed in *ABCC6* knockdown HepG2 cells may also explain the gene dysregulation found in both the previous study [20] and the present one. Accordingly, the modulation of the activity of several transcription factors by thiol redox state has been extensively reviewed. Redox regulation of DNA–protein interaction is an important step through which transcription factors can control gene transcription. Crucial cysteine residues have been discovered in the DNA-binding domain of several transcription factors and found to modulate their ability to bind DNA [33].

Furthermore, de Boussac et al. showed a correlation between ROS levels and ABCC6 expression, demonstrating that oxidative stress induced by vitamin K3 and tert-butyl-hydroquinone inhibits ABCC6 expression [34]. The decrease in lamin A/C expression found in *ABCC6* knockdown cells may impinge on gene expression profiles, as shown for other pathophysiological conditions associated with lamin A dysregulation [35]. As a consequence, *ABCC6* knockdown HepG2 cells, which show a characteristic gene expression profile, could also adopt an altered secretory phenotype that may modify the tissue microenvironment, as already shown for several types of senescent cells [36, 37] including senescent hepatocytes [38]. Concurrent with this hypothesis, a recent study shows that abrogated ABCC6 function causes alterations in the metabolic profile of the liver, which aligns with the concept of PXE being a metabolic disease originating from liver disturbance [39].

## Conclusions

All findings show that the knockdown of *ABCC6* changes the cellular phenotype and suggest that the previously reported change in ATP secretion [15] is only one of the effects of ABCC6 deficiency. In this scenario, the PXE syndrome might be considered a complex metabolic disorder not related to the mere absence of PPi in the plasma.

## Abbreviations

ABC: ATP binding cassette
ACDC: Arterial calcification due to deficiency of CD73
CD73: Cluster of differentiation 73
DCC: Dystrophic cardiac calcification
GACI: Arterial calcification of infancy
GSH: Reduced glutathione
GSSG: Oxidized glutathione
OPN: Osteopontin
PXE: Pseudoxanthoma elasticum
ROS: Reactive oxygen species
SA-β-gal: Senescence-associated β-galactosidase
TNAP: Tissue nonspecific alkaline phosphatase
